# Thermal Ablation of Liver Tumors Guided by Augmented Reality: An Initial Clinical Experience

**DOI:** 10.3390/cancers14051312

**Published:** 2022-03-03

**Authors:** Marco Solbiati, Tiziana Ierace, Riccardo Muglia, Vittorio Pedicini, Roberto Iezzi, Katia M. Passera, Alessandro C. Rotilio, S. Nahum Goldberg, Luigi A. Solbiati

**Affiliations:** 1 R&D Unit, R.A.W. Srl, 20127 Milano, Italy; m.solbiati@endo-sight.it (M.S.); k.passera@endo-sight.it (K.M.P.); a.rotilio@endo-sight.it (A.C.R.); 2 Department of Radiology, IRCCS Humanitas Research Hospital, Rozzano, 20089 Milan, Italy; t.ierace@tin.it (T.I.); vittorio.pedicini@humanitas.it (V.P.); 3 Department of Radiology, ASST Papa Giovanni XXIII, 24127 Bergamo, Italy; rmuglia@asst-pg23.it; 4 Department of Diagnostic Imaging, Oncologic Rediotherapy and Hematology, IRCCS Policlinico Universitario A. Gemelli, Università Cattolica del Sacro Cuore, 00168 Rome, Italy; roberto.iezzi.md@gmail.com; 5 Department of Radiology, Hadassah Hebrew University Medical Centre, Jerusalem 90221, Israel; sgoldber@bidmc.harvard.edu; 6 Department of Biomedical Sciences, Humanitas University, Pieve Emanuele, 20089 Milan, Italy

**Keywords:** augmented reality, three-dimensional (3D) reconstruction, interventional oncology, computed tomography, liver

## Abstract

**Simple Summary:**

We report the first clinical use of Endosight, a new guidance system for percutaneous interventional procedures based on augmented reality, to guide percutaneous thermal ablations. The new system was demonstrated to be precise and reliable, with a targeting accuracy of 3.4 mm. Clinically acceptable, rapid setup and procedural times can be achieved.

**Abstract:**

Background: Over the last two decades, augmented reality (AR) has been used as a visualization tool in many medical fields in order to increase precision, limit the radiation dose, and decrease the variability among operators. Here, we report the first in vivo study of a novel AR system for the guidance of percutaneous interventional oncology procedures. Methods: Eight patients with 15 liver tumors (0.7–3.0 cm, mean 1.56 + 0.55) underwent percutaneous thermal ablations using AR guidance (i.e., the Endosight system). Prior to the intervention, the patients were evaluated with US and CT. The targeted nodules were segmented and three-dimensionally (3D) reconstructed from CT images, and the probe trajectory to the target was defined. The procedures were guided solely by AR, with the position of the probe tip was subsequently confirmed by conventional imaging. The primary endpoints were the targeting accuracy, the system setup time, and targeting time (i.e., from the target visualization to the correct needle insertion). The technical success was also evaluated and validated by co-registration software. Upon completion, the operators were assessed for cybersickness or other symptoms related to the use of AR. Results: Rapid system setup and procedural targeting times were noted (mean 14.3 min; 12.0–17.2 min; 4.3 min, 3.2–5.7 min, mean, respectively). The high targeting accuracy (3.4 mm; 2.6–4.2 mm, mean) was accompanied by technical success in all 15 lesions (i.e., the complete ablation of the tumor and 13/15 lesions with a >90% 5-mm periablational margin). No intra/periprocedural complications or operator cybersickness were observed. Conclusions: AR guidance is highly accurate, and allows for the confident performance of percutaneous thermal ablations.

## 1. Introduction

Precision and targeting accuracy are key for the success of all image-guided interventional procedures. Over the last 20 years, several new navigational tools have been added to conventional imaging modalities (ultrasound, CT, MRI) with the purpose of increasing precision, favouring dose reduction, decreasing variability among operators, and thus promoting the diffusion of diagnostic and therapeutic interventional procedures based on ever-increasing reliability. Image fusion platforms based on electromagnetic or optical devices [1,2,3,4], CT with laser marker systems [5], CT fluoroscopy [6], cone-beam CT [7,8], CT with electromagnetic tracking [9], and robotic systems [10] have been incorporated into clinical practice in many centers. However, these tools still have some limitations, such as the inability to provide a real, live, 3D visualization of the target and the surrounding structures, the need for the operator to alternate their gaze between the interventional field and the instrumentation screen(s), a steep learning curve, and, for CT-guided procedures, potentially substantial radiation doses to patients and operators [11]. Recently, spatial computing technology has allowed the development of simulated reality environments, virtual reality (VR) and augmented reality (AR), which enable real-time interaction by the user. VR completely immerses the user in an artificial, digitally created 3D world through head-mounted displays (HMDs), with the user having no direct interaction with the real world. Therefore, in the medical field, VR can be used for surgical planning and simulation, but not for the direct guidance of interventional procedures [11,12]. To the contrary, AR overlays digital content onto the visualized real world through an external device [12,13,14], enhancing reality with superimposed information, using optical see-through head-mounted displays (HMDs or “goggles”), screens, smartphones, tablets and videoprojectors, such that digital and physical objects are visualized simultaneously. This permits their interaction with each other, thus allowing guidance of interventional procedures. The capability for computers to enhance visibility and navigate through 3D coordinates during minimally invasive interventional procedures was first noted in 1997 [15]. Since then, AR has been clinically applied as a visualization tool to augment anatomical [16] and pathological structures in neurosurgery [17,18,19] and vascular [20,21], orthopedic [22,23], urologic [24,25,26], plastic [27], and abdominal surgery [28,29]. This was achieved by creating 3D anatomic volumes from cross-sectional scans or angiographic images, and manually overlapping them over patients positioned in the real operating field [3] through electromagnetic or optical tracking systems and computer vision algorithms. In Interventional Oncology, AR was initially tested on phantoms to assist with percutaneous biopsies [30,31], and subsequently for the assessment of its potential role for the augmentation of minimally invasive surgery for the accurate localization of organ, or the guidance of radiofrequency ablation (RFA) or irreversible electroporation (IRE) electrodes on phantoms [32,33], but not for the direct guidance of interventional procedures in humans. To our knowledge, this is the first report of the targeting and ablation of small hepatic malignancies in human patients using AR as the sole modality of guidance.

## 2. Materials and Methods

This study was performed at two tertiary referral centres for liver diseases (Humanitas Research Hospital and IRCCS Policlinico Universitario A. Gemelli), with the approval of the local Institutional Ethics Committees. Written informed consent was obtained from all of the subjects involved in the study.

### 2.1. Patient Population

Fifteen hepatic malignancies (9 hepatocellular carcinomas (HCCs), 3 metastases from breast carcinoma, and 3 from pancreatic adenocarcinoma) in eight patients (5 males and 3 females, median age 72.5 years, range 56–83) underwent AR-guided percutaneous thermal ablation. The treated nodule size ranged from 0.7 to 3.0 cm (mean 1.56 + 0.55).

For all of the cases, the treatment decision was determined by the consensus of an Institutional Multidisciplinary Liver Team. According to the BCLC classification, the nine HCCs in five patients were either very early (8/9 cases) or early stage (1/9), in a subset of HCV-related early stage cirrhosis (Child-Pugh A, ECOG PS 0) [34]. These were located in segments VIII (*n* = 4), V (*n* = 2), II (*n* = 2) and VI (*n* = 1); the sizes ranged from 1.2 to 3.0 cm (mean 1.69 + 0.53). One patient had four HCCs, and one two HCCs. All of the nodules were treated in the same session. The other three patients had only one HCC. All of the HCCs were diagnosed through a non-invasive radiological work-up, following the European Association of the Study of the Liver (EASL) 2018 clinical practice guidelines [35].

The six metastases in the three patients ranged from 0.7 to 2.1 cm (mean 1.35 cm + 0.56) in size, and were diagnosed by percutaneous US-guided biopsies using 20 G Menghini-modified needles (Sterylab, Milan, Italy).

### 2.2. Pre-Treatment Diagnostic Assessment

**Figure 1:**
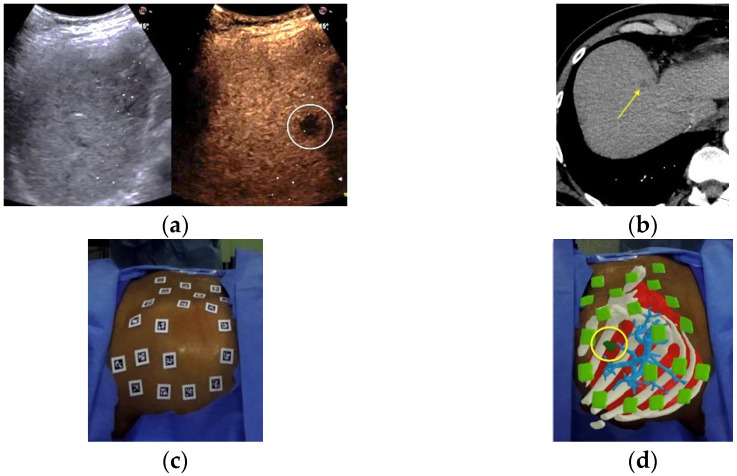

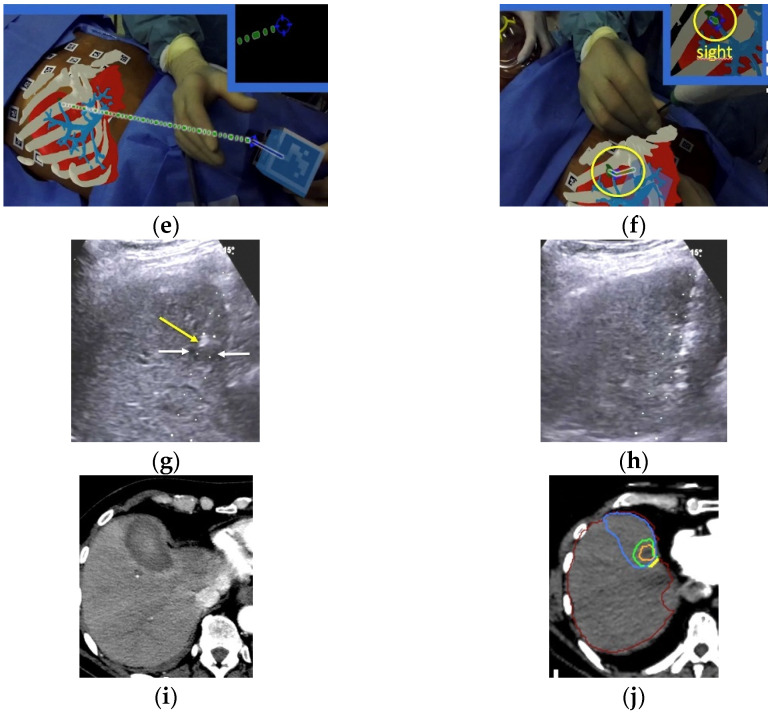
Augmented reality guided ablation: a 1.5-cm pancreatic carcinoma metastasis at segment VIII, poorly visible on B-mode US and clearly seen by CEUS (**a**), and seen on pre-ablation CT scan (arrow) (**b**). Radiopaque markers with no repetitive pattern applied to the patient’s skin (**c**). View through the operator’s HMD: ribs (in white), major hepatic blood vessels (light blue), liver (red), and target lesion (green, in a yellow circle) (**d**). View through the HMD, showing that the operator can see the virtual needle (blue line) and the line that connects the tip of the needle to the center of the target (in green) (**e**). Following the trajectory line permits successful tumor targeting with AR guidance alone (**f**). The 5.4-mm distance between the tip of the coaxial needle and the target center by US (**g**). Subsequently, the microwave antenna is inserted into the coaxial needle (**h**). On a post-ablation CT scan, a large ablation volume completely surrounds the metastasis (**i**). Using ablation confirmation software (Ablation-fit^TM^), the technical success achieved was precisely demonstrated. The margins of the target tumor are shown in orange, the 5-mm ablation margin is shown in green, and the margins of the necrosis volume are shown in blue. Complete tumor ablation with only 5.4% of the safety margin out of the necrosis volume was achieved (**j**).

All of the patients were initially evaluated with a baseline ultrasound of the liver which included contrast enhanced ultrasound (CEUS) after the intravenous administration of 2.4 to 4.8 mL second-generation contrast agent (SonoVue, Bracco, Milan, Italy) (Figure 1A), and an abdominal contrast enhanced computed tomography (CECT) in the arterial, portal, and late phases (Figure 1B). In order to achieve registration for the orientation reference of the AR display, twenty radiopaque markers with no repetitive pattern were applied to the abdominal skin in the right hypochondrium surrounding the area of interest (Figure 1C) immediately prior to the treatment. A new CECT in the arterial and portal phases was acquired during free breathing (i.e., normal respiration), paying particular attention to include all of the markers within the scanning area. In 14 of the 15 patients, CT scans were acquired with two different machines (Ingenuity, Philips Healthcare, Cleveland, OH, USA for 4 patients, and Revolution EVO, General Electric, Boston, MA, USA for 3 patients) following the injection of Iopamidol (Iopamiro 370, Bracco, Milan, Italy) at 4 mL/s, using a 2-mm slice thickness, a matrix of 512 × 512 pixels, an in-plane pixel size of 0.48–0.78 mm, 1:1 pitch, 120 kVp and 180 mA. In the last patient, 70 mL Iomeprol (Iomeron 400 mg/mL, Bracco, Milan, Italy) was injected at 3 mL/s using Lightspeed VCT 64 (General Electric, Boston, MA, USA) using a 2.5-mm slice thickness, a matrix of 512 × 512 pixels, an in-plane pixel size of 0.48–0.78 mm, 1:1 pitch, 120 kVp and 180 mA.

### 2.3. Augmented Reality Settings

**Figure 2:**
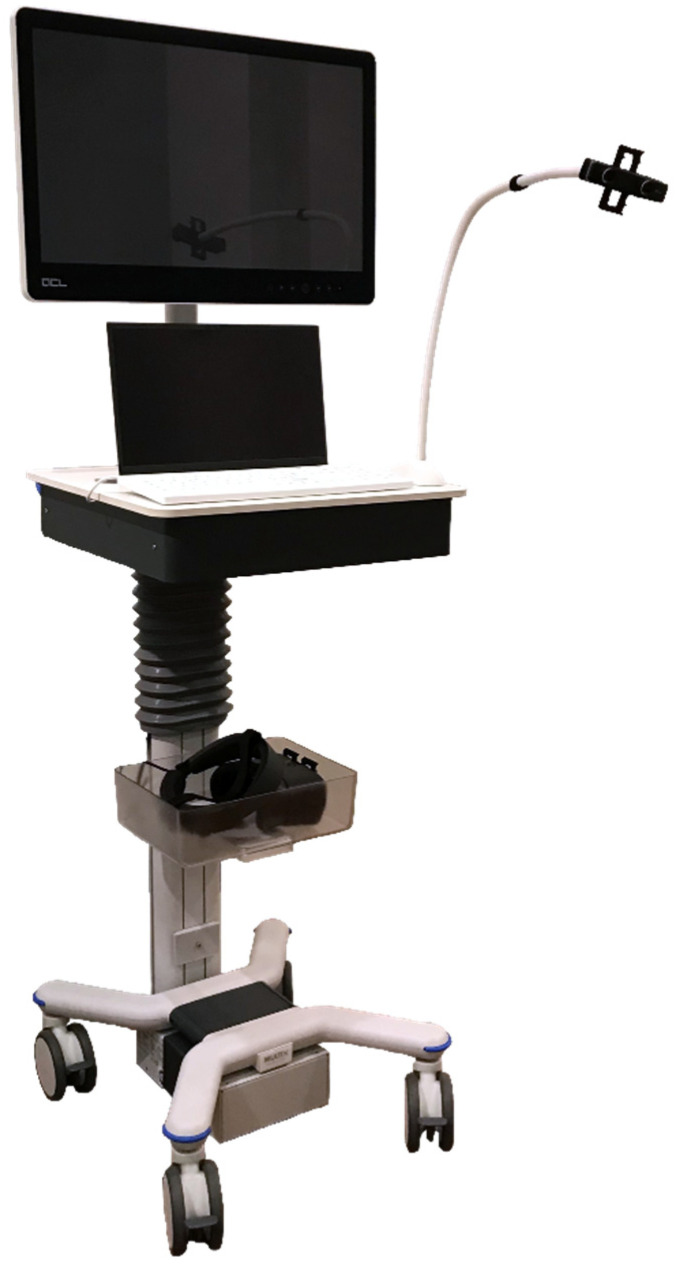
Endosight system overview: cart, medical display, laptop, and Oculus Rift-S paired with a Zed Mini camera.

The AR set-up comprised a proprietary augmented reality system (Endosight, R.A.W. Srl, Milan, Italy) that features a 27” medical display (ACL, Leipzig, Germany), a laptop (Dell Technologies, Round Rock, TX, USA) with installed proprietary image processing and augmented reality software, and a commercially available head-mounted display (HMD) (Oculus Rift-S, Facebook Technologies, Menlo Park, CA, USA) paired with a binocular camera (Zed Mini, Stereolabs, San Francisco, CA, USA) (Figure 2).

The binocular camera viewed the patient from two different angles in order to register the patient model in the camera frame using the markers visible in both video images while tracking the ablation applicator. The software enabled the 3D reconstruction (from CT scans to 3D volumes), co-registration, and AR intervention. Specifically, after uploading the CECT scans into the system, followed by the automatic segmentation and 3D reconstruction of the liver, spleen, bones, liver blood vessels and radiopaque markers, the semi-automatic segmentation of the target lesions occurred using proprietary reconstruction algorithms. In addition, the most suitable trajectory path from the skin to the target was defined. Subsequently, by moving the HMD around the patient, the system software co-registered (matched) all of the radiopaque markers segmented on the CT scans with all of the real markers applied to the patient’s skin. This allowed for the simultaneous visualization of the patient’s surface and internal anatomy, the target lesion, and the trajectory path to the target in 3D, by superimposing—in real-time—virtual images on the operator’s real field of sight (Figure 1D). Next, in order to allow the visualization of the probe position during the procedure, a clip with five markers with no repetitive pattern was attached to either a 14 G (for 14 ablations) or a 11 G (for one ablation) coaxial needle, 7.8 cm in length (Bard Inc., Murray Hill, New York, NY, USA), that was used as a coaxial ablation device introducer (Figure 1E).

### 2.4. Treatment Procedure

**Figure 3:**
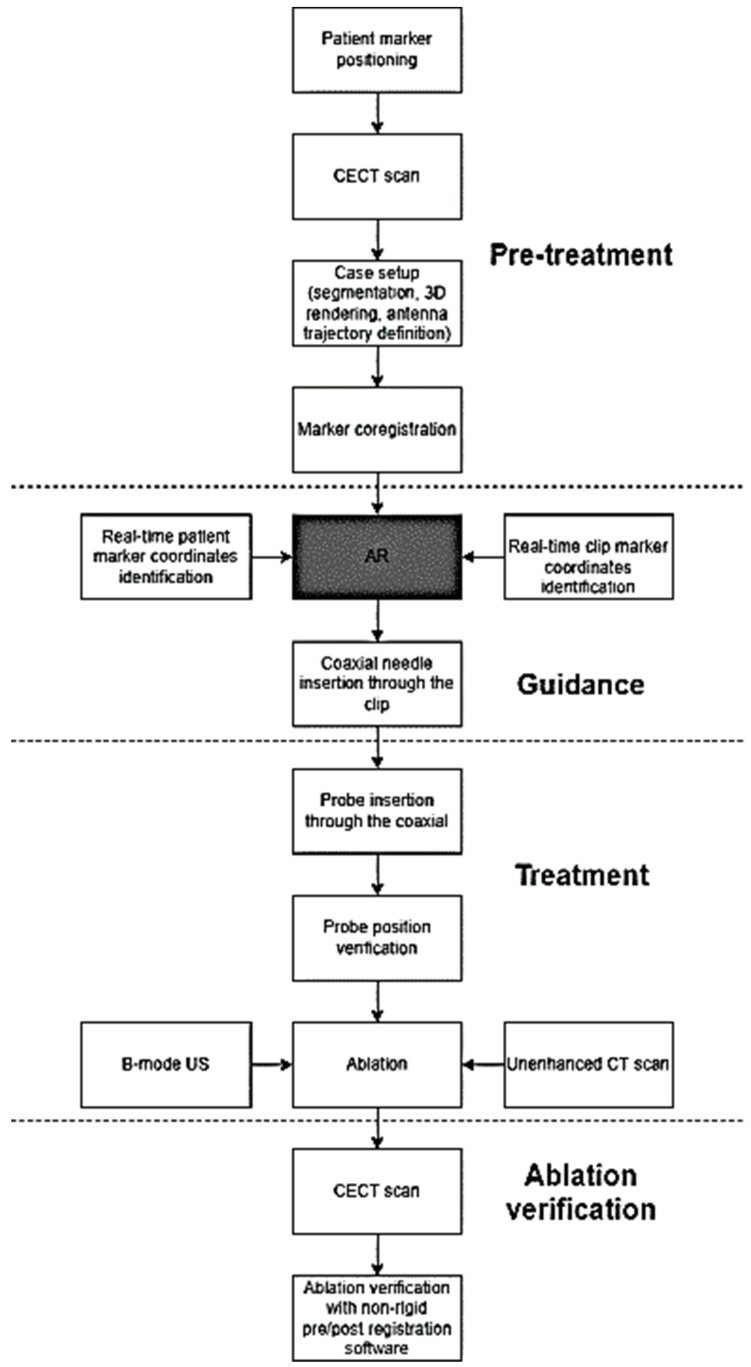
Workflow of the AR-guided thermal ablations.

All of the procedures were performed by three interventional radiologists with more than 15 years of experience in percutaneous thermal ablations. In 14 of the 15 patients, the ablations were performed in the CT room coupled with real-time ultrasound, under assisted ventilation, during short-acting anaesthesia using propofol (AstraZeneca, Cambridge, UK) (10 mg/mL) and alfentanil (Hameln Pharma, Gloucester, UK) (0.5 mg/mL), with continuous hemodynamic monitoring throughout the procedure. In the remaining patient, the ablation was performed under direct CT control (Lightspeed VCT 64) after local anesthesia and deep sedation with 0.2 mg Fentanyl (Janssen-Cilag, Beerse, Belgium) without additional ultrasound guidance. Using AR guidance alone, the coaxial needle was inserted following the predefined trajectory line planned during the setup (Figure 1F). This was facilitated by color coding, in that when the predefined trajectory line overlapped the virtual needle line, this path turned from blue to green in the AR visual field, highlighting and denoting the correct alignment. The insertion was conducted during the patient’s free breathing (as in the pre-ablation acquisition of the CT scans) in order to minimize the organ displacement caused by breathing. The depth from the entry point (i.e., the skin) to the target centre was measured in real-time by the software, and was visualized on the operator’s HMD. Before the introduction of the ablation device into the coaxial needle, the position of the coaxial needle and its correspondence with the real location of the target nodule was verified using real-time US when the target nodule was visible with US, or with CT when the target was invisible on US. In order to assess the precision of the AR, the distance from the real target centre visualized on the US or CT and the virtual target centre shown by the trajectory line starting from the tip of the coaxial needle was measured (Figure 1G). The ablation probe was then inserted, positioning its tip 5–7 mm beyond the deep margin of the target in order to achieve sufficient ablative margins (Figure 1H). Then, the coaxial needle was partly retracted while maintaining the positioning of the ablation device in order to achieve the complete exposition of the active tip. Microwave ablations (MWA) were performed with 13 G, 15 cm-long antennae (Medtronic, Dublin, Ireland) for three malignancies of three patients, and 14 G, 15 cm-long antennae (HS Hospital Service, Aprilia, Italy) for eleven nodules of five patients. The remaining patient recieved RFA performed with a 14 G, 15 cm-long electrode with a 3-cm exposed tip (RF Medical, Seoul, Korea). The treatment power and duration, and the total amount of energy delivered were selected based upon the size and location of each nodule, according to the device manufacturer’s technical recommendation and operator experience. Figure 3 shows the complete treatment procedure workflow.

### 2.5. Post-Procedural Assessment

The CECT was performed immediately after withdrawing the ablation device (Figure 1I). A proprietary ablation-confirmation software (Ablation-fit^TM^, R.A.W. Srl, Milan, Italy) [36]—which enables the automatic segmentation of the liver and intrahepatic blood vessels, and semi-automatically co-registers the target nodules on pre-ablation CT scans with the volumes of necrosis achieved on post-ablation scans using a non-rigid registration tool—was used in order to assess the precision and completeness of the ablation volume achieved (Figure 1J). Using a 3D model, the software verified whether the volume of ablative necrosis included entirely or partially the tumor and a pre-defined ablative margin (5-mm thick, in these cases), as well as quantifying, as a percentage, the amount of tumor and ablative margin (if any) external to the ablation volume, thus allowing us to assess the technical success of the procedure [37,38]. After the ablation, all of the operators were interviewed regarding the need for manual adjustments of the HMDs and the occurrence of eye fatigue, dizziness, or cybersickness.

### 2.6. Statistical Analysis

The primary endpoints evaluated included the time required to set up the system and to position the antenna tip inside the nodule, the mean depth of the target centre from the needle entry point on the skin, and the deployment accuracy, defined as the mean distance between the geometric center of the target and the ablation device tip measured on unenhanced CT or US. Secondary endpoints included the technical success, i.e., the complete ablation of the entire tumor and the achievement of an >90% 5-mm periablational margin ablation [36], complications, and operator sensations regarding the procedure. The data were analyzed with statistical software (SPSS, version 17.0), and were reported as the mean ± standard deviation (SD), or as the mean and range.

## 3. Results

The time required to set up the system ranged from 12.0 to 17.2 min (12.3 ± 2.1 min), and the time required to perform each insertion and tumor targeting ranged from 3.2 to 5.7 min (4.3 ± 0.9 min). In 7 of the 15 (46.7%) cases, the target nodule was visible on the US, and the real location of the target nodule and the position of the coaxial needle tip in respect to the target centre were verified using real-time US. In the remaining 8 of the 15 (53.3%) cases, unenhanced CT was employed for verification. The mean depth of the target centre from the needle entry point on the skin was 76.0 ± 28.2 mm. The distance between the geometric center of the target and the ablation device tip measured on unenhanced CT or US ranged from 2.1 to 4.5 mm (3.2 ± 0.7 mm). Table 1 shows—for each target—the size, the distance of the interventional device tip from the tumor center, the time taken to reach the target, and the modality used for the verification.

For the MWA, the power delivered ranged from 50 to 60 W, with a treatment duration of 5 min in four HCCs, and 6 min in the remaining five HCCs and the five metastases. For the case of radiofrequency ablation (RFA), the power delivered was 1500 mA for 12 min. A single ablation device insertion was performed for each target tumor. Technical success was achieved in each case. After the automatic coregistration of the 3D volumes of the pre-ablation tumors and post-ablation necrotic changes, achieved with the Ablation-fit^TM^ software, the complete ablation of the tumors (i.e., no residual unablated portion of the target tumors) was found. The residual 5-mm ablative margin percentage ranged from 0 to 14.1 % (5.5 ± 4.3%), with 13 of the 15 (86.7%) patients showing >90% ablation of this margin. Table 2 shows the residual 5-mm safety margin (in percentage) of each target lesion.

No intra- or periprocedural adverse events occurred. No user-dependent calibration and adjustment for the HMD was needed, and no significant eye fatigue or “cybersickness” was reported by any of the users.

## 4. Discussion

Modern imaging modalities enable the visualization of increasingly small target lesions, often in difficult-to-target locations, which is particularly suitable for local, image-guided treatments (IGTs). Consequently, the requests for image-guided therapy, accompanied by expectations of favorable outcomes, are constantly increasing. However, some problems still remain unsolved. First of all, the learning curve for the use of these technologies is often long, and this limits the diffusion of interventional procedures, particularly among young operators and/or in low-referral centers. The lack of the real, live, 3D visualization of targets, and the poor working ergonomics (the need to check many screens simultaneously, restricted line-of-sight to screens, and the need to alternate the operator’s gaze between the interventional field and the instrumentation screens) are additional important limitations. The mental registration of the target position seen in the reference image (US, CT, MRI) with the corresponding position in patients is often challenging, particularly for liver dome lesions requiring non-orthogonal or out-of-plane approaches, even when CT guidance is used. The difficulty and subjectivity of this process may also increase the risks for patients. Thus, the need for a technically easy combination of “real-world” visualization with virtual objects precisely superimposed upon the scene is increasingly desired. This can be achieved with AR technology in the actual interventional field, where the operator can visualize and interact simultaneously with the real world (patient, interventional instrumentation) and virtual objects (hidden organs and targets, surrounding structures seen on CT and MRI, etc.) based on the superimposition of the “two worlds”, as displayed on HMD, smartphones, tablets, screens or videoprojectors. Moreover, HMDs can be relatively advantageous compared to the direct line of sight through the lens display [39].

The most critical issue for the use of AR in medical applications is the superimposition precision, i.e., the registration accuracy. Multiple studies on phantoms, animal models, and human cadavers have primarily focused upon the assessment of registration accuracy, either for AR navigation [40] or the AR guidance of needles [19,31,32,33,41,42]. Hecht et al. [41] reported a smartphone-based AR system for needle trajectory planning and real-time guidance on phantoms. In their first experiment, the mean error of the needle insertion was 2.69 + 2.61 mm, which was 78% lower than the CT-guided freehand procedure. In their second experiment, the operators successfully navigated the needle tip within 5 mm on each first attempt under the guidance of the AR system, which eliminated the need for further needle adjustments. In addition, the procedural time was 66% lower than the CT-guided freehand procedure. Long et al. [42] compared the accuracy and the placement time needed by five interventional radiologists and a resident with a range of clinical experience (3–25 years) to place biopsy needles on millimetric targets positioned in an anthropomorphic abdominal phantom at different depths, using cone-beam CT (CBCT)-guided fluoroscopy, and smartphone- and smartglasses-based AR navigation platforms. The placement error was extremely small and virtually identical for all of the three modalities (4–5 mm), and the placement time was significantly shorter for smartphones and HMDs (38% and 55% respectively) than for CBCT. Additionally, the results were achieved by AR without intra-procedural radiation, and with a learning curve of only 15 min.

Using the same system employed for the present study, Solbiati et al. recently published a proof-of-concept study on phantoms, animal models, and human cadavers targeted with AR guidance. In the rigid phantom, sub–5-mm accuracy (2.0 + 1.5 mm) (mean + standard deviation) was achieved. In a porcine model with small (2 × 1 mm) metallic targets embedded, the accuracy was 3.9 + 0.4 mm when the targeting was performed with respiration suspended at maximum expiration, as in the initial CT scan, and 8.0 + 0.5 mm when the procedure was performed without breathing control. In a human cadaver attached to a ventilator to induce simulated respirations, two liver metastases (1.8 cm and 3.0 cm) were targeted with an accuracy of 2.5 mm and 2.8 mm, respectively [43].

Here, we note the similar accuracy of 3.4 mm in living, breathing patients. Regarding AR-guided needle insertions in human patients, De Paolis et al. [32] reported their preliminary experience in locating a focal liver lesion in the operating room just before open surgery. The surgeon was able to determine the correct position of the real tumor by touching it and applying the ablation applicator to it in order to verify the correct overlap between the virtual and the real tumor. Although an excellent accuracy of 2 mm was reported, problems of depth perception and instrument visibility occurred whenever the surgeon’s body was located between the tracker and the instrument, both of which related to the use of the optical tracker.

The AR system used for our current report is specifically designed to guide percutaneous biopsies and ablation procedures. It is based on disposable markers with no repetitive pattern affixed to the patient’s abdominal skin before performing the CT scans. The associated software enables us to visualize and segment the markers on the patient (virtual objects) and the target tumor, to automatically register and superimpose virtual and real images in real-time, to define the safe and accurate trajectory line to the target center, to depict the guided movements of the interventional device without the need for additional imaging, and to show the whole procedure on a display, HMD, or screen [43,44]. The main advantage of HMD is the 3D visualization, which tops the 2D visualization of smartphones and tablets. The results achieved were very promising: the accuracy of the antenna tip with respect to the center of the target was well below the 5-mm threshold (with a mean of 3.2 + 0.7 mm), and technical success of the ablation was achieved in all cases. The mean times required to set up the system and to perform each insertion were 14.3 min and 4.3 min, respectively, and were independent of the type of ablation system used. This is not substantially different from the time usually required to perform CT-guided procedures by expert radiologists, even after a long learning curve. Moreover, the software for the assessment and quantification of the tumor ablation margins in 3D was integrated into the AR system, enabling the immediate and accurate evaluation of the technical success [36].

In recent years, two issues have been raised regarding the technology of HMDs used for AR, i.e., the field of view (FOV) and the need for calibration [39]. The binocular FOV of human eyes is naturally about 200 o in the horizontal plane and 135 o in the vertical plane, while commercially available HMDs had initial FOVs ranging around 30–40 o. This limitation has recently increased to 90 o both horizontally and vertically. Nevertheless, the calibration of HMDs is needed to tailor projections to the user’s interpupillary distance. Given that most HMDs have fixed focal planes, when the calibration is inaccurate, the eyes can focus and converge at separate distances, causing distorted depth perception, eye fatigue and “cybersickness” due to discrepancies between the visual and vestibular senses. Nowadays, commercially available HMDs are provided with two videocameras, which has eliminated the need for user-dependent calibration and adjustment. This has limited the common occurrence of the cybersickness which was reported previously, as noted in our study.

The patient’s respiratory movement and motion remain one of the largest technical and practical hurdles, as AR guidance systems are currently unable to follow respiratory excursions in mobile organs with real-time corrections, bearing a risk of the shifting of the intended target relative to the expected location. Other target-related limitations arise from the abilities of lesions to warp or move within their environments. Respiratory motion tracking and the monitoring of respiration during deep sedation or general anesthesia seem to offer the best solutions to date. The guiding information is provided regularly at the point of the breathing that matches the respiratory phase during which the preoperative CT image was acquired (the middle respiratory or expiratory phase). In this time interval window, the operator can move the needle toward the target as rapidly as possible. In our study, the insertion was conducted during the patient’s free breathing (as in the pre-ablation acquisition of the CT scans) in order to minimize the organ displacement caused by breathing. Given that this was the initial study of AR-guided thermal ablation, we selected only tumors which were visible on US or on CT in order to be able to check the position of the device tip after its insertion, before starting the ablation. Probe repositioning was never required, as the position achieved with AR guidance was always sufficiently accurate. Nevertheless, we acknowledge that this will not always be invariable, and note that—should minor placement corrections be needed—the virtual system will potentially save a substantial amount of radiation exposure compared to fully CT-guided procedures, be they CT-guided freehand, cone-beam CT, or CT fluoroscopy guidance [20]. Indeed, in the experimental study conducted by Park et al. [39] comparing a HoloLens-based 3D AR-assisted navigation system with CT-guided simulations, the AR system reduced the radiation dose by 41%.

An additional potential challenge of AR-guided interventional procedures is needle bending during the insertion, exacerbated by increased applied pressure or the use of thinner needles. The solution we successfully utilized was the use of a rigid coaxial needle to maintain the interventional device fixed in 3D space during its advancement, minimizing the bending of the ablation device inserted into the coaxial needle. We further demonstrated that the attachment of a clip with markers with no repetitive pattern to the coaxial needle permits precise monitoring by AR of the probe advancement towards the target, and the interventional device subsequently inserted into the coaxial needle can easily hit the target center. Coaxial needles have been used for interventional procedures for decades, and do not appreciably increase the risk of bleeding because their construction is engineered to result in an ultimate size only 1–2 G larger than that of ablation devices or biopsy needles.

With respect to other navigation systems, AR guidance offers an ergonomic advantage that the overlay of treatment information (anatomy, target, trajectory line, etc.) is shown directly in the procedural environment, and not on a display screen away from the patient on a monitor, as occurs with CT- or CBCT-guided fluoroscopy. Additional advantages of AR guidance are the ease of use, the reduced procedural time compared to more traditional guidance systems, and the short learning curve (compared to that of CT-guided procedures), which is particularly useful for young operators with limited experience, who perform equally or even better than senior operators with long experience. Furthermore, AR guidance systems are significantly less expensive than all of the other needle guidance systems. This may favour the diffusion of AR, and consequently of image-guided procedures in small centers, and in developing countries that cannot afford to buy complex and expensive guidance technologies (the so called “democratization” of interventional procedures).

With AR, the same images seen by the operator can also be visualized on monitors inside and outside the interventional room, and can be broadcast on a larger scale, allowing interventional radiologists to visualize live or recorded procedures performed by experts. AR can provide not only an excellent opportunity for physician training and education but also a very useful tool to exchange collaborative experiences among various centers for remote real-time instruction or expert assistance [12,45].

We acknowledge that this study has some limitations, most notably the small number of patients within the cohort, and the non-randomized type of lesions treated, all of which visible on both US and CT despite their small size. Nonetheless, we believe that it will encourage new prospective studies, and will work as the basis for the development of AR technology in the clinical field.

## 5. Conclusions

In this retrospective study, we obtained high standards of targeting accuracy, technical efficacy, procedural time, and radiation dose reduction using AR as the sole guidance method for percutaneous thermal ablation, without encountering any complications. In spite of the small cohort analyzed, we propose that our preliminary data demonstrate the potential for AR, with further validation, to become a leading and low-cost modality for the guidance of interventional procedures worldwide.

## Figures and Tables

**Table 1:** Sizes of the targets, the distance of the interventional device tip from the center of each target tumor, the time needed to reach the target, and the modality used for the distance measurement.

	Size [mm]	Distance from Target Center [mm]	Time to Reach Target [min]	Modality Used for Measurement
Patient 1—Target 1	1.8	3.1	3.3	US
Patient2—Target 1	1.8	3.8	4.1	US
Patient 3—Target 1	1.5	2.1	5.7	CT
Patient 3—Target 2	1.7	2.4	3.2	CT
Patient 3—Target 3	1.4	3.6	4.9	CT
Patient 3—Target 4	1.2	2.7	4.2	CT
Patient 4—Target 1	1.4	3.9	5.3	US
Patient 4—Target 2	1.4	2.9	3.4	US
Patient 5—Target 1	2.1	3.6	5.3	CT
Patient 6—Target 1	1.8	2.4	4.0	CT
Patient 6—Target 2	0.8	2,2	4.2	CT
Patient 7—Target 1	3.0	4.5	5.2	CT
Patient 8—Target 1	1.5	4.1	3.3	US
Patient 8—Target 2	1.2	3.1	3.6	US
Patient 8—Target 3	0.7	3.4	4.9	US
Overall:	1.56 ± 0.55 mm	3.2 ± 0.7 mm	4.3 ± 0.9	

**Table 2:** Residual 5-mm safety margin (as a percentage) of each target tumor, calculated by the Ablation-fit^TM^ software.

	Residual 5 mm Safety Margin [%]
Patient 1—Target 1	5.4
Patient 2—Target 1	2.8
Patient 3—Target 1	3.1
Patient 3—Target 2	9.2
Patient 3—Target 3	12.1
Patient 3—Target 4	1.9
Patient 4—Target 1	0
Patient 4—Target 2	4.9
Patient 5—Target 1	8.1
Patient 6—Target 1	14.1
Patient 6—Target 2	10.1
Patient 7—Target 1	4.1
Patient 8—Target 1	3.3
Patient 8—Target 2	3.1
Patient 8—Target 3	0

## Data Availability

The data presented in this study are available on request from the corresponding author.
